# Professional Freedom

**Published:** 1907-11-23

**Authors:** 


### EDITOR'S LETTER-BOX.

**[Our Correspondents are reminded that prolixity is a great bar to publication, and that brevity of style and conciseness of statement greatly facilitate early insertion.]**

### "PROFESSIONAL FREEDOM."

[Mr. Robbins, of the "National Anti-Vivisection Hospital," has favoured us with yet another letter, and in this he writes: "I am only too glad to inform you in so many words that the medical and surgical staff of this hospital are *not* 'forbidden to make use of some kinds of treatment.'" We have made no pretence to decide the issue of fact between the hospital in question and the Hospital Sunday Fund. The latter, in refusing a grant to the Anti-Vivisection Hospital, stated that "By the rules of your Governing Body the Distribution Committee understand your medical officers are forbidden to make use of some kinds of treatment which, in the opinion of the highest authorities in medicine in all parts of the civilised world, have been considered to possess valuable curative powers. The Lay Governing Body, therefore, imposes restrictions as to treatment on its medical staff." In the official correspondence which followed this statement the authorities of the Anti-Vivisection Hospital did not contest the above proposition, but pleaded that "The Governing Bodies of most hospitals impose restrictions on their medical applicants"; and Mr. Robbins, in his previous communications to us, was equally silent on the point. We now print his somewhat belated denial, and our readers must judge for themselves between this and the affirmation of the Hospital Sunday Fund.—ED. HOSPITAL.]

